# Association between walking 5000 step/day and fall incidence over six months in urban community-dwelling older people

**DOI:** 10.1186/s12877-020-01582-z

**Published:** 2020-06-05

**Authors:** T. Aranyavalai, C. Jalayondeja, W. Jalayondeja, S. Pichaiyongwongdee, J. Kaewkungwal, J. J. Laskin

**Affiliations:** 1 Faculty of Physical Therapy, Mahidol University, 999, Phuttamonthon 4, Road, Nakhon Pathom, Salaya District 73170 Thailand; 2 Faculty of Medicine Vajira Hospital, Navamindradhiraj University, Bangkok, Thailand; 3 Department of Tropical Hygiene, Faculty of Tropical Medicine, Mahidol University, Bangkok, Thailand; 4 School of Physical Therapy & Rehabilitation Sciences, University of Montana, Montana, USA

**Keywords:** Falls, Physical activity, Older people, Walking

## Abstract

**Background:**

Walking is the most common population-wide campaign for health promotion in older people. However, the cutoff threshold for walking steps/day to identify the older people who are at risk of falling is not recommended. Therefore, the objectives were to investigate the association between all possible risk factors including physical performance, physical activity and fall incidence over the six-month in community-dwelling older people who had low-risk of falling and to identify walking threshold (steps/day) for reducing risk of fall.

**Methods:**

The older people who aged ≥60 years and had free of falling for 1 year were invited to participate in this study. They lived in five communities in Bangkok Thailand. Demographics and physical performances were collected at baseline. Walking (step/day) and 24-h physical activity (PA) were monitored for 5 consecutive days by the Actical® accelerometer wrapped on non-dominant wrists. The Physical Activity Scale for the Elderly (PASE) questionnaire was used to record activities in the past 7 days by interview. A monthly calendar was used to record fall incidence over the 6 months. Unadjusted and adjusted hazard ratio (HR) with 95% confidence interval (CI) were analyzed using the Cox’s proportional hazard regression. The Kaplan Meier curve illustrated the probability to survive from fall over the 6 months.

**Results:**

Of 255, 33 older people (12.94%) reported first-fall incidence over the 6 months. Fall incidence density rate was 0.79 per 1000 person-day. Our findings showed that significant association between fall incidence and behavioral risk factors including PASE scores < 100 (HR = 3.53; 95% CI: 1.24–10.04), walking < 5000 steps/day (HR = 3.6; 95% CI: 1.76–7.31) and moderate to vigorous intensity of PA at < 60 min/week (HR = 3.66; 95% CI: 1.12–12.01). Fall incidence were related to the following risk factors: age (HR = 3.54; 95% CI: 1.37–9.11), took polypharmacy/antipsychotics (HR = 4.32; 95% CI: 2.12–8.79), presence of urinary incontinence (HR = 2.87; 95% CI: 1.45–5.68), low functional mobility by Timed Up and Go ≥13.5 s (HR = 6.43; 95% CI: 2.65–15.57).

**Conclusions:**

This study proposed walking ≥5000 steps/day as a cutoff threshold to recommend for reducing risk of falling in community-dwelling older people who had low-risk of falling.

## Background

Falls are the most common cause of death among older people worldwide. The World Health Organization (WHO) reports that the worldwide prevalence rate for falling vary from 28 to 35% annually among older people [1]. In Southeast Asia, the number of fallers range from 10.4 to 53.6% in those aged ≥60 years [2]. Specifically, in Thailand, the prevalence for falls was highest amongst older people living in urban and suburban communities, 18.7 to 19.8% respectively [3, 4]. These falls were responsible for 11.0% of deaths in the Thai older population [5]. Consequently, the risk factors predisposing older Thai people to falls should be investigated and identified.

The potential fall risk factors in older people have been identified and include: age, gender, living environments, health status and medical conditions. In 2007 the WHO proposed categorizing these risk factors into four dimensions; biological, behavioral, environmental and socioeconomic [1–4, 6]. Of these potential factors, insufficient physical activity (PA) has been defined as an important preventable risk factor for falls in older people. The WHO and American Collage of Sport Medicine (ACSM) recommends that older people should engage in moderate-to-vigorous physical activity (MVPA) for at least 30 min/day for 5 or more days a week and accumulated in bouts of at least 10 min. Older people should also include some form of balance training and resisted exercise program at least 2 days/week [7, 8]. These activity recommendations not only to reduce the risks for a number of common chronic diseases but also enhance muscle strength and endurance which may facilitate fall prevention in older people.

Walking is frequently regarded as the most feasible and accessible PA that could increase lower extremity muscle strength, improve balance performance and psychological conditions [9]. Population-wide walking health promotion campaigns have been utilized across the lifespan. Tudor-Locke et al. reviewed many previous studies and proposed the public health guideline for walking steps/day versus accumulated minutes of PA for reducing the risk of all-cause mortality in older people. To meet the recommendation goal, the older people encourage to walk 7000 to 10,000 steps/day to provide the health benefit equivalent to MVPA for 30 min/day [10]. Marshall et al. suggested that moderate-intensity walking should performed by older people at a pace of at least 100 steps/min and accumulating 3000 steps/day or 30 min/day at least 5 days/week [11]. This walking prescription can also be used to meet the WHO’s recommendation for health promotion.

However, a minimum cutoff threshold for walking steps/day to reduce falling in older people is unclear. Many previous studies have investigated the efficacy of walking programs on fall prevention in older people but the results are varied [12–16]. Okubo et al. demonstrated the impact of a walking at a self-selected pace for 30 min, 2–3 times a week. They found that fall occurrence decreased over the 16-month follow-up in older people who participated in the walking program when compared to those received only balance training [13]. Sherrington et al. examined the benefits of a walking and balance training program on fall reduction in older people with high-risk of falling [14]. A high-risk of falling is defined as when an individual demonstrates 2 or more fall risk factors including age ≥ 75 years and having impaired muscle strength and/or a previous fall [15, 16]. They found that walking program was ineffective among older people with high fall risk falling. In fact, a walking program could actually increase the experience of fall injury among older inactive people. Any recommendation for the addition of a walking prescription into a fall prevention program must take into account of the individual’s current fall risk.

Given the poor understanding and limited research into what is the walking threshold for reducing fall occurrence and the association of fall risk factors and PA in older people it is essential that this work be pursued. Therefore, the primary objective of this study was to investigate the association between typical fall risk factors including physical performance, PA and fall incidence over a six-month period in community-dwelling older people who have a low-risk of falling. The secondary objective of this study was to identify walking threshold (steps/day) for reducing risk of fall.

## Methods

This six-month prospective observational study was conducted to monitor fall incidence among older people who lived in five urban communities at Bangkok, Thailand. Individuals who aged 60 years and over were invited to participate in this study. Inclusion criteria included: (a) no falls within the past 12 months, (b) able to walk outdoors independently with or without a one-point cane, (c) good cognition and communication measured by the Thai Mini-Mental State Examination (TMMSE> 22/30 and > 17/30 for literate and nonliterate participants) [17] and (d) had corrected visual acuity measured by a Snellen chart [18]. In addition, the participants in this study must have been classified as having a low-fall risk according to the Falls Risk for Older People in the Community (FROP-Com < 19 of 60 scores) [19] and Modified Falls Efficacy Scale (MFES≥112 of 140 scores) [20]. Participants were excluded if they had serious medical conditions including orthopedic, neurologic and cardiovascular conditions that restricted the functional mobility including walking and activity in daily living.

This study was approved by the Mahidol University Institute Research Board (MU-CIRB COA. NO. 2018/079.0404). The participants received information of study’s protocol and data collection before signed the inform consent to participate in this study.

Data was collected from May 2018 to January 2019. The first 3 months were a recruitment period and baseline assessments were completed. Demographics collected at baseline included: personal characteristics (age, gender, marital status, body mass index (BMI), level of education, and monthly income.), health status and medical conditions by FROP-Com and living environment status. Eligible participants were divided into 12 cohorts and each cohort contained 18 to 24 participants. At baseline their physical performance and physical activity were assessed by physical therapists. Then the first-fall incidence over the 6 months was individually monitored using a self-report monthly calendar. The total person-day at risk of falling was recorded as time to the first-fall within 6 months among fallers and time to the end of study 6 months among non-fallers.

### Fall incidence

Fall incidence was a primary outcome in this study. The first fall episode was recorded during the six-month of follow-up. Fall was defined as *“inadvertently coming to rest on the ground, floor, or other lower level, excluding intentional change in position to rest in furniture, wall or other objects”* [1]. This definition was clearly explained to all participants and their caregivers at baseline. Participants were instructed to record their first fall episode, cause of falling and its consequences in the fall monthly calendar and submit this information to the village health volunteer (VHV). The reported consequences of falling comprised of severity of injury (none/minor/severe), body area injury, type of treatments (none/medication/surgery), and fall location (indoor or outdoor, at home or other places). The causes of falling included slip, trip, poor lighting, bump or ran into an object. The VHVs in each community visited the participant’s home and gathered monthly calendar information every 2 weeks.

### Physical performance tests

At baseline, physical performance tests were assessed by three physical therapists. Physical performance tests comprised of five times sit to stand (FTSTS), the Berg Balance Scale (BBS), timed up and go (TUG) test, ten-meter walk test (10mWT) and six-minute walk test (6MWT). Excellent inter-rater reliability of these tests was noted [21]. FTSTS is an outcome measure to quantify lower extremity muscles strength. The time of FTSTS was recorded (cut off score > 13 s referred to high-risk of falling) [22]. The BBS contained 14 basic activities to measure static and dynamic balances in older people. The cutoff score for BBS < 45 of 56 was commonly used to identify those who had risk for falling [23]. The TUG test was used to assess gait and functional mobility [24]. Older people who performed TUG ≥13.5 s were classified into high-risk for falling group [25]. The 10mWT is widely used to measure the gait speed [26]. The older people who walked slower than ≤0.6 meters/second were classified as high-risk of falls [27]. The 6MWT is used for assessing the exercise capacity and cardiovascular endurance test. The distance for Thai’s normative reference values equal 256.3 to 366.1 meters for women and 306.6 to 389.6 meters for men [28]. Those who less then these reference values were considered to be at a high risk for falling.

### Walking steps/day and physical activity

Walking steps/day and caloric expenditure were measured by the Actical® accelerometer (Philips Respironics, Bend, OR, USA) worn for 5 consecutive days. One week prior to the baseline physical performance testing, an Actical® accelerometer was placed on the participant’s nondominant wrist using nylon wrist band. It was positioned on the dorsal aspect of the wrist, just proximal to the radial and ulnar processes. These devices are water proof and were worn 24 h/day. Before testing, the accelerometers were calibrated and set to record data over 15-s epochs. The Actical® accelerometers were set to start for recording the data at midnight (12.00 AM) after donning the device. Data from 12 h per day (from 6.00 AM. to 6.00 PM) were selected as the minimum amount of time needed to identify a valid day. All participants were requested to carry out their usual activities for the 5 consecutive days of monitoring by the accelerometers. The Actical® accelerometer has been validated to capture PA behavior in various populations [29–33]. It provides valid estimates of step counts at self-selected paces and walking behaviors of community-dwelling older [31, 32]. In our study, we calculated energy expenditure based on the regression equations from Heil’s et al. [29]. The total walking steps/day, total calorie expenditure and the amount of time for PA on week days and weekend days (sedentary (SB), light (LPA) and moderate-to-vigorous intensity of physical activity MVPA) were recorded. In addition, the daily MVPA, in at least 10-min bouts, was calculated and analyzed. Based on the WHO’ recommendation for PA in older people, MVPA> 150 min/week and > 60 min/week were determined for data analysis. Based on Tudor-Locke’s studies [34–36], they divided walking steps/day into four level for identifying level of physical activity in older people: < 2500 steps/day (basal activity), 2500–4999 steps/ day (limited activity), 5000–7499 steps/day (low active), 7500–9999 steps/day (somewhat active), and ≥ 10,000–12,499 steps/day (active). They indicated walking < 5000 steps/day as the sedentary lifestyle cutoff for older people. Therefore, this study selected the cutoff for walking < 5000 steps/day for determining high risk of falling in older people.

When the participants returned the accelerometers they were interviewed their physical activity during the past 7 days using the Physical Activity Scale for the Elderly (PASE). The PASE is commonly used to determine level of PA in community-dwelling older people. PASE comprised of 13 items and completed by interview administration for 10 min. The intra-rater reliability has been reported to be high (ICC = 0.75, 95% CI = 0.69–0.80) [37]. PASE score was calculated from weights and frequency values of 12 items including walk outside home, sport/recreational activities with light/moderate/strenuous effort, strengthening and endurance exercise, light and heavy housework, home repairs, lawn work or yard care, outdoor gardening, caring for another person and work for pay or as volunteer. The total scores ranged from 0 to 360. According to Washburn et al. [37], PASE < 100 scores defined as low level of PA for the older people who aged 70 years.

### Statistical analysis

As the primary objective of this study, we aimed to determine the six-month first fall incidence and its relationship to the assessed baseline. We will estimate the fall incidence rate over the 6 month follow up period and hypothesized that those who had high physical activity levels would have a low fall incidence. Therefore, we selected the sample size formula for proportion based on Wayne [38]. The sample size (n) equals [Z_α_^2^ p(1-p)]/d^2^. The type I error (α) was set at 0.05 with 5% of marginal error (d). Based on the previous study, the prevalence of fall (p) was 20% in Thai older people living in urban area [4]. Therefore, the total number of sample size was 246.

Data were analyzed using the SPSS® (version 19.0; IBM, Armonk, NY). Descriptive statistical analysis was used for baseline data (i.e, demographics, physical performances, walking and physical activity). The independent t-test was used for comparison baseline data between fallers and non-fallers if the data was normally distributed. For nonnormality data, the Mann Whitney U test was used. The significance level was set at *p* < 0.05. The probability of fall occurrence during the six-months was expressed in terms of a fall incidence density rate. The fall incidence density rate is the number of first-fall episodes within the 6 months divided by the total person-day at risk. The person-day is an estimation of the actual day at risk of falling. We summed the days of observation which started from participant’s enrollment to the date of first-fall event (fallers) or to the end of study of 6 months (non-fallers) or to the date of withdrawal or death (censored).

In this analysis of fall risk factors, the main analysis was focusing on factors related to the physical performance and physical activity measured at baseline including: FTSTS, BBS, TUG, 10mWT, 6MWT, PASE, number of steps/day, and MVPA and the occurrence of a fall over the 6-month follow-up period. The Cox’s proportional hazard model was performed in two steps. First the model fit fall incidence with potential risk factors including personal characteristics (age, gender, BMI), health and medical condition (FROP-Com, medical conditions) and living status. The factors that showed statistical significant (*p* < 0.05) were considered potential confounding factors and used for adjustment in the second step. Age, polypharmacy/psychotics drugs, medical condition and urinary incontinence are potential confounders in this analysis. In the second step, the model fit the fall incidence with main factors of interest (i.e., physical performances and physical activities) with and without the selected potential confounding factors found in step 1. Unadjusted and adjusted hazard ratio (HR) with 95% confident interval (CI) were calculated by the Cox’s proportional hazard regression. This study illustrated the fall incidence as the function of time by the Kaplan-Meier (KM) curve. It showed the survival function of falling during the 6 months among older people and classified by walking step per day. The log rank test was used to compare the survival function of fall among older people who walk ≥5000 steps/day and < 5000 steps/day.

## Results

A total of 933 older people living in five communities of Bangkok were invited to participate in this study. Of these 295 individuals volunteered to participate and of these 255 people met the inclusion criteria. Their ages ranged from 60 to 89 years and 183 persons were female (71.8%). They lived in Masajid Bansomdet community (*n* = 85), Wat Noi Hirunrujee community (*n* = 60), Prasanmit community (*n* = 50), Wat Yai Srisuphan community (*n* = 40) and Sri Phum community (*n* = 87). Forty persons were excluded because they had cognitive impairment (*n* = 16), poor visual acuity (*n* = 22) or used a walker when outside the home (n = 2).

Table 1 presents demographics, physical performance and physical activity among those included in the study. The results showed that 33 people reported a fall (fallers) and 222 did not (non-fallers) overt the 6 month follow-up period. Significant differences of age, FROP-Com, FTSTS, BBS, TUG, 10mWT, 6MWT, walking (steps/day) and PA between faller and non-faller groups were observed at baseline. Fallers demonstrated low scores of PA including self-reported measure by PASE and accelerometer.

**Table 1:** Demographics, physical performance tests and physical activity among older people

Baseline characteristics	Total (*n* = 255)	Fallers (*n* = 33)	Non-fallers (*n* = 222)	*p-*value
Demographics				
Age (years)	68.7 ± 6.7	71.4 ± 7.4	68.3 ± 6.5	0.03^*^
BMI (kg/m^2^) ^a^	24.3 ± 3.9	24.7 ± 3.5	24.2 ± 4.1	0.49
FROP-Com (scores)	7.81 ± 3.3	10.4 ± 3.9	7.4 ± 3.1	< 0.001^**^
MFES (scores)	136.3 ± 3.7	134.2 ± 4.6	136.6 ± 3.5	0.69
Physical performance tests				
FTSTS (second)	13.1 ± 2.3	14.9 ± 2.3	12.8 ± 2.2	< 0.001^**^
BBS (scores)	50.2 ± 3.3	48.1 ± 3.8	50.5 ± 3.2	< 0.001^**^
TUG (second) ^a^	12.3 ± 2.5	14.8 ± 2.5	12.4 ± 2.4	< 0.001^**^
10mWT (meter/second)	0.7 ± 0.2	0.6 ± 0.1	0.8 ± 04	< 0.001^**^
6MWT (meter)	308.6 ± 48.1	278.2 ± 45.9	313.1 ± 46.8	< 0.001^**^
Physical activity^b^				
PASE (scores)	75.3 ± 47.9	48.54 ± 36.9	79.45 ± 48.1	< 0.001^**^
Accelerometer				
Walking (step/day)	6694.6 ± 3386.9	4677.1 ± 2970.4	7005.7 ± 3345.9	< 0.001^**^
Sleeping (minute/week.)	2193.3 ± 122.4	2232.7 ± 112.01	2187 ± 123.03	0.08
Sedentary (minute/week.)	927.63 ± 185.05	1007.5 ± 128.5	915.32 ± 189.5	< 0.001^**^
LPA (minute/week.)	86.7 ± 115.1	38.5 ± 45.4	94.1 ± 120.6	< 0.001^**^
MVPA (minute/week.)	51.7 ± 80.7	20.9 ± 49.4	56.4 ± 83.6	< 0.001^**^
MVPA for 10 min bouts (minute/week.)	34.9 ± 73.6	17.3 ± 52.1	37.6 ± 76.2	< 0.001^**^
Energy estimated activity (kcal/week.)	242.5 ± 187.9	171.3 ± 117.1	253.5 ± 194	< 0.001^**^

Abbreviations. *FROP-Com* Falls Risk for Older People in the Community (score 0–60), *MFES* Modified-Falls Efficacy Scale (score 0–140), *PASE* The Physical Activity Scale for Elderly (score 0–360), *LPA* Light intensity of physical activity, *MVPA* Moderate to vigorous intensity of physical activity, *kg/m*^*2*^ kilogram/meter^2^, *kcal* kilocalories

^a^Data analysis using the independent t-test

^b^Eight persons did not completed 5 days of physical activity monitoring by Actical® accelerometer (*n* = 247)

^*^p < 0.05 and ^**^p < 0.001

**Fig. 1:**
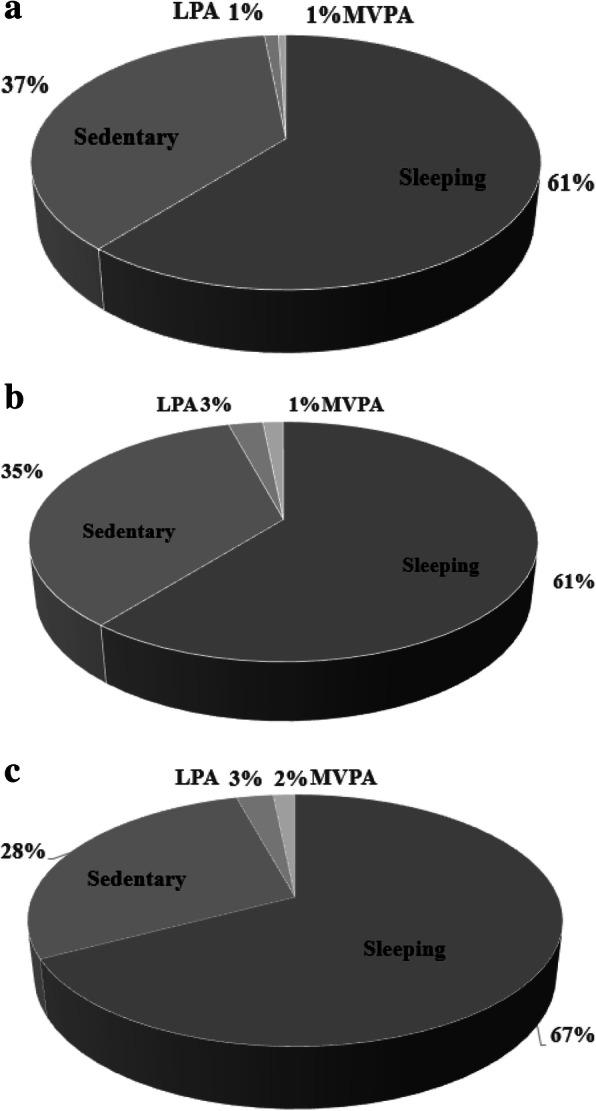
Physical activity (minute/week) including sleeping, sedentary behavior (SB), light physical activity (LPA) and moderate-to-vigorous physical activity (MVPA) measured by the Actical® accelerometer: **a** fallers, **b** nonfallers and **c** total

Figure 1 illustrates the proportion of time spent during the day 24-h including: sleeping, sedentary behaviors (SB), light physical activity (LPA) and moderate-to-vigorous physical activity (MVPA) measured by the Actical® accelerometer. MVPA was performed approximately 30 to 60 min per week in total, fallers and non-fallers.

### Fall incidence and hazard ratio (HR) according to fall-risk factors over the 6-month

Of a total 255, 33 participants (12.9%) reported a fall over the six-months and their total time at risk was 41,656 person-day. The fall incidence density rate was 0.79 per 1000 person-day or 0.79 per 1000 cases fell over 1 day of observation. The reported falls occurred inside the house (45.5%), outside the house (12.1%) and in community (42.4%). The reported consequences of the falls were bruising or skin abrasions (*n* = 16), extremity joint sprains (*n* = 14), fractured neck of femur (n = 1), or no symptoms (*n* = 2).

The results demonstrated a significant association between fall incidence and fall-risk factors including age and fall risk factors measured by FROP-com: polypharmacy/psychotics drug, medical conditions and urinary incontinence (Table 2). High risk of falling was demonstrated in older people aged 80–89 years when compared with age < 80 years. The older people who had taken polypharmacy/psychotics drug, ≥3 medical conditions and urinary incontinence were higher risk of falling than those who did not.

**Table 2:** Fall incidence and hazard ratio (HR) according to the fall-risk factors over the 6-month in older people (*n* = 255)

Fall risk factors	Total (n = 255)	Fallers (*n* = 33)	Person-day (days)	Incidence density (per 1000 person day)	HR_unadj_	95%CI	*p*-value
Age group (years)							
80–89 years	21	6	3013	1.99	3.54	1.37–9.11	0.001^*^
70–79 years	74	12	12,006	0.99	1.78	0.83–3.79	0.14
60–69 years	160	15	26,637	0.56	1.00		
Gender							
Female	183	24	29,990	0.80	1.04	0.48–2.23	0.92
Male	72	9	11,666	0.77	1.00		
BMI (kg/m^2^)							
Obese (BMI ≥ 25)	98	15	15,948	0.94	1.66	0.67–4.10	0.27
Overweight (23.0 ≤ BMI < 25.0)	65	8	10,811	0.74	1.47	0.55–3.96	0.44
Normal (18.5 ≤ BMI < 23.0)	76	8	12,359	0.65	1.00		
Lean (BMI < 18.5)	16	2	2538	0.79	1.39	0.30–6.70	0.68
FROP-Com							
Polypharmacy/psychotics drug							
Yes	34	12	4944	2.43	4.32	2.12–8.79	0.001^*^
No	221	21	36,712	0.57	1.00		
Medical conditions							
≥ 3 conditions		11	4955	2.21	3.75	1.82–7.74	0.001^*^
0–2 condition		22	36,701	0.60	1.00		
Urinary incontinence							
Yes	74	17	11,361	1.49	2.87	1.45–5.68	0.003^*^
No	181	16	30,295	0.53	1.00		
Living alone							
Yes	29	2	4801	0.41	0.48	0.13–1.99	0.39
With family	226	31	36,855	0.84			
Income per month (baht)^a^							
≤ 2600 baht	134	18	21,565	0.83	1.11	0.56–2.21	0.76
> 2600 baht	121	15	20,091	0.75	1.00		

^a^ classified from median of income/month

Table 3 demonstrates the association between physical performances, physical activity and fall incidence over the 6-month in older people. For the crude analysis of fall risk factors, unadjusted hazard ratio (HR_unadj_) was expressed as greater than 1. High risk of fall demonstrated in older people who had FTSTS> 13 s, BBS ≤ 45, TUG> 13.5 s, 10mWT ≤ 0.6 meters/second, 6MWT < 306 meters, PASE< 100 scores, walking< 5000 steps/day and MVPA< 60 min/day. After adjusted for potential confounders, TUG≤13.5 s and walking< 5000 steps/day were significantly related to fall incidence over 6 months (*p* < 0.05). The older people who walk < 5000 steps/day were 2.62 times more likely to fall than those who walk ≥5000 steps/day (*p* < 0.009).

**Table 3:** The relationship between physical performances and physical activity and fall incidence over the 6-month in older people (n = 255)

Fall risk factors	Total (n = 255)	Fallers (n = 33)	Person-day (days)	Incidence density (per 1000 person day)	Cox-proportional hazard model					
					HR_unadj_	95%CI	*p*-value	HR_adj_	95%CI	*p*-value
FTSTS										
> 13 s.	148	26	23,339	1.11	2.92	1.27–6.73	0.01^*^	1.02	0.36–2.91	0.97
≤ 13 s.	107	7	18,317	0.38	1.00					
BBS										
≤ 45 scores	20	7	2859	2.44	3.67	1.59–8.46	0.002^*^	1.27	0.45–3.61	0.66
> 45 scores	235	26	38,797	0.67	1.00					
TUG										
> 13.5 s.	142	27	17,189	1.57	6.43	2.65–15.57	0.001^*^	4.22	1.69–10.56	0.002^*^
≤ 13.5 s.	57	6	24,467	0.25	1.00					
10mWT										
≤ 0.6 m/s	57	17	8579	1.98	4.13	2.08–8.17	0.001^*^	1.01	0.42–2.39	0.98
> 0.6 m/s	198	16	33,077	0.48	1.00					
6MWT										
< 306 m	138	27	21,453	1.26	4.25	1.75–10.29	0.001^*^	1.99	0.68–5.83	0.21
≥ 306 m	117	6	20,203	0.30	1.00					
PASE										
< 100 scores	169	29	28,027	1.03	3.53	1.24–10.04	0.02^*^	1.44	0.47–4.43	0.53
≥ 100 scores	78	4	13,629	0.29	1.00					
Accelerometer										
Walking										
< 5000step/day	84	21	13,570	1.55	3.60	1.76–7.31	0.001^*^	2.62	1.27–5.42	0.009^*^
≥ 5000step/day	163	12	28,086	0.43	1.00					
MVPA										
< 150 min/wk.	227	31	38,261	0.81	1.37	0.33–5.75	0.66	1.44	0.47–4.43	0.53
≥ 150 min/wk.	20	2	3395	0.59	1.00					
< 60 min/wk.	183	30	30,477	0.98	3.66	1.12–12.01	0.03^*^	1.55	0.42–5.76	0.51
≥ 60 min/wk.	64	3	11,179	0.27	1.00					

Adjusted by age, polypharmacy/psychotics drugs, medical condition and urinary incontinence

### The Kaplan-Meier curve of fall incidence according to walking steps/day

**Fig. 2:**
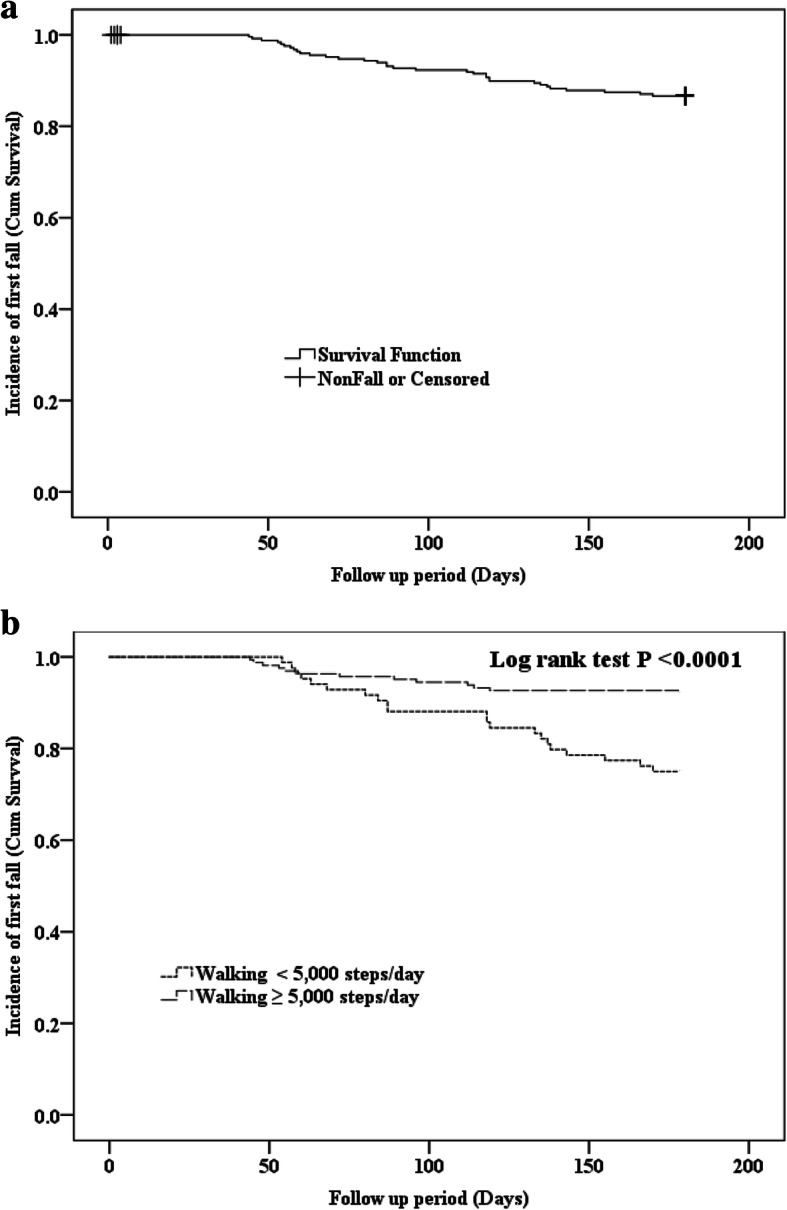
A Kaplain-Meier curve illustrating the fall incidence among older people: (**a**) total subjects and (**b**) subjects who walked <5000 and ≥ 5000 steps/day

Figure 2 demonstrates the probability of fall occurrence over the 6 months in total older people and those who classified in walk ≥5000 steps/day and < 5000 steps/day. Based on the survival analysis and KM curve, the overall survival rate of fall was 87.06% among older people who had low-risk of falling. The median time to survive from fall was 44 days after baseline. Significant different of probability of fall occurrence over the six-month between older people who walking ≥5000 steps/day and < 5000 steps/day (*p* < 0.001) (Fig. 2).

## Discussion

This study established the six-month prospective observational fall incidence among community-dwelling older people who had low-risk of falling. This prospective study presents reliable data which provides a novel look at the association between a wide variety of fall risk factors and the fall incidence in older individuals who were at a low risk for falls [39]. Our results showed that 33 participants (12.94%) reported a falls over the six-month follow-up period. The incidence density rate of fall was 0.79 per 1000 person-day which represent the significant of fall issue in older people who had low-risk of falling. Therefore, the attribute risk factors of falling should be avoided.

Our results demonstrated a significant association between fall incidence and demographics, physical performances, walking step/day and physical activity. Personal characteristics including age and medical conditions were identified as risk of falling in older people who had low-risk of falling. Older people who aged over 80 years were 3.54 times more likely to fall than those who younger. If they took polypharmacy/psychotics drug, had at least 3 or more medical conditions and had urinary incontinence, the risk of falling was higher than those who did not. Our results are point out in the same direction as the previous’s findings [40, 41]. Deandrea et al. [40] reported that older people who took sedative, antipsychotics and antidepressant drugs, had medical conditions, particularly urinary incontinent were more likely to fall than those who did not. However, they found marked heterogeneity findings among studies and various strength of association between fall and risk factors (the odds ratio ranged from 0.88–2.00). Lawlor et al. [42] indicated the population attribute risk of falling increased 32.2 to 50% when the older people reported having at least one chronic condition or taking at least one psychotic drug. They indicated that chronic illness such as urinary incontinence, diabetes, and arthritis can causes of limit physical activity which led to increased risk of falling among older people [42].

At baseline, the results demonstrated that non-fallers showed better physical performance than those who fell. Decreased lower extremity muscle strength, poor balance, altered gait and functional mobility can be the cause of a trip or slip or fall in older people [43, 44]. Therefore, these essential components should to be considered for fall prevention programs in older people. We selected five standardized tools with their cutoff score for determining fallers over the 6 months. Our results demonstrated that the older people whose TUG was < 13.5 s were more likely to fall over the 6 months than those who had TUG of ≥13.5 s. After adjusted for potential confounders, the TUG showed a significant association with fall incidence over the 6 months (HR = 4.22, 95%CI 1.69–10.56, *p*-value = 0.002). Our findings are in agreement with previous studies that used the TUG for identifying the risk of falling among community-dwelling older people [25, 45, 46]. Shumway cook et al. [25] proposed the TUG with a cutoff score 13.5 s for identifying community-dwelling older people who are prone to falls. The TUG is a sensitive and specific measure (sensitivity = 87% and specificity =87%) and can account for 90% of the overall fall prediction. Consequently, this study’s findings support that the TUG could be a robust and valid tool for identifying community dwelling older people who are at risk for falls.

Our results showed a significant association between walking and fall incidence over the 6 months (*p* < 0.009). We hoped to identify a walking threshold (steps/day) for reducing the risk of falls in low fall risk older people. Typically, the guidelines recommended for walking only addresses the health and low risk of all-causes mortality in older people. There is a lack of information that identifies the walking steps/day for reducing the risk of falling. Tudor-Locke et al. stated that the normative data of walking step/day ranged from 2000 to 9000 steps/day among healthy older people [10, 34–36]. To transfer the public health guideline of walking steps/day, they estimated the number of step count by multiply the adult cadence of 100 steps/min with 30 min/day. However, identifying of step-defined sedentary lifestyle index, the number of walking step should over and above 3000 steps/day. They proposed walking< 5000 steps/day as a cutoff threshold for sedentary lifestyle in older people. Okubo et al. [9] reported that fall incidence did not increase over 12 weeks in the older people who received walking program 30-50 min/day for 3–5 day/week. Their walking increased from 6156.7 ± 3046.1 steps/day at baseline to 9448.6 ± 3324.6 steps/day at 12-week. Walking has specific effect on improve fall-related physical factors measured by Fall Efficacy Scale. They suggested that walking might be useful as population-wide recommended approach for fall prevention among general community-dwelling older people.

Based on these studies, we selected the cutoff threshold for walking at < 5000 steps/day as the sedentary lifestyle index to identify high risk of falling in older people in this study. After considering age, polypharmacy/psychotics drugs, medical condition and urinary incontinence, the older people walking< 5000 steps/day were more likely to fall than those who walk ≥5000 steps/day. In addition, the risk of falling was reduced more than 60% in those who walked ≥5000 steps/day (HR_adj_ = 0.38, 95%CI = 0.14–0.63, *p* = 0.002). Therefore, our study supported that walking < 5000 steps/day as the cutoff threshold to identify the older people who are at risk of falling. Walking 5000 steps/day is attainable and practicable in older people. Health care practitioners can disseminate through a public health campaign for reducing risk of falling in community-dwelling older people who had low-risk of falling.

A couple of unexpected outcomes were demonstrated in our study. First, the results showed a nonsignificant association between MVPA and fall incidence over the 6 months in community-dwelling older people. The recommendation of MVPA< 150 min/week or < 60 min/week could not identify the older who are at risk of falling over the 6 months. This might be a result of the small number of participants who performed a MVPA of ≥150 min/week (*n* = 22) in this study (Fig. 1). Therefore, faller and non-fallers could not be differentiated by MVPA. However, our study demonstrated that a lower time and calorie expenditure of MVPA in fallers when compared to non-fallers (*p* < 0.001). Second, the PASE < 100 scores could not identify the older people who fell. It is possible that the older people engaged in PA with no steps or minimal steps such as cycling or gardening. PA is a complex behavior to measure accurately at the population level, it is possible to obtain useful information based on PASE, provided the right subjective measurement instruments are chosen and used correctly [37].

This study had many strengths as compared to other works. First, we conducted a prospective observational study of fall incidence over a six-month period. The prospective study presents more reliable data which can contribute significantly to fall research [39]. This study is a robust validation study which identified the risk factors of falls in community-dwelling older people who had low-risk of falling. This study evaluated the PA level and fall incidence by the valid tools. PA and physical performance was measured using both subjective and objective measurements which significantly reduced the error of outcome measures. Lastly, we recorded fall incidence using a monthly calendar that was monitored every 2 weeks. This methodology helped ensure accurate fall event detection that reduced a common source of error.

Some limitation of this study was the low number of participants who met the PA recommendation of MVPA≥150 min/week (*n* = 22), this was unexpected as they were defined as having a low-risk of falling. We recommend for future study to identify the MVPA min/week and its relationship to fall occurrence to examine not just the quantity of PA but also the intensity if habitual activity that may help prevent falls.

## Conclusion

This study established the prospective six-month fall incidence among older people who are at a low-risk of falling. The results of this study demonstrated that fall incidence density rate was 0.79 per 1000 person-day or 0.79 per 1000 cases fell over 1 day of observation. The potential risk factors for falling including age, polypharmacy/psychotic drug, several medical conditions and urinary incontinence were identified at the first analysis of fall risk factors. After adjusted for these potential confounders, walking< 5000 steps/day was a significant risk factors of falling in older people (*p* < 0.05). In addition, the older people who walked ≥ 5000 steps/day were less likely to fall. Therefore, we support the cutoff threshold for walking ≥ 5000 steps/day to reduce risk of fall incidence in older people who have a low-risk of falling. This information could help to support a population-wide campaign for reducing falls and preventing fall related injuries or deaths in older people. Walking 5000 steps/day is an optimal goal for health care practitioners to communicate the strategy of reducing risk of fall in community-dwelling older people who had low-risk of falling.

## Data Availability

On reasonable demand, the corresponding author was supply the datasets used and/or analyzed the study.

## Abbreviations

WHO: World Health Organization
ACSM: American Collage of Sport Medicine
MVPA: Moderate-to-Vigorous Physical Activity
HR: Hazard ratio
CI: Confidence interval
RR: Relative risk
TMMSE: Thai Mini-Mental State Examination
FROP-Com: Falls Risk for Older People in the Community
MFES: Modified Falls Efficacy Scale
BMI: Body Mass Index
VHV: Village health volunteers
FTSTS: Five times sit to stand
BBS: Berg balance scale
TUG: Timed up and go test
10mWT: Ten meters walk test
6MWT: Six minutes walk test
ICC: Intraclass correlation efficiency
OR: Oregon
USA: The United Stated Of America
PA: Physical activity
PASE: Physical Activity Scale for the Elderly
AM: Ante meridiem
PM: Post meridiem
SB: Sedentary behavior
LPA: Light intensity of physical activity
Kcal: Kilocalorie
NY: New York
KM: Kaplan Meier
wk.: Week
N: Number
